# American Institute of Homœopathy

**Published:** 1899-05

**Authors:** Benj. F. Bailey

**Affiliations:** President; Lincoln, Nebraska


### AMERICAN INSTITUTE OF HOMŒOPATHY.

LINCOLN, NEBRASKA, March 17th, 1899.

EDITOR HOMŒOPATHIC PHYSICIAN, PHILADELPHIA, PA.:

During a recent visit to Chicago, Washington, Philadelphia, New York, Boston, and Atlantic City, it was my privilege to attend the annual meeting of the Executive Committee of the American Institute of Homœopathy in New York, to meet the local Committee of Arrangements at Atlantic City, and to come in touch with the profession generally.

The Executive Committee arranged a programme which will give every section at least one meeting before the entire Institute. There will be seven papers on the different fields of Homœopathy. There will be special features of unusual interest. The memorial exercises will be held during a recess in a busy session, and will be of a character to command our respect and cause us to indeed realize the solemnity of the occasion. It will be a session of thoroughly scientific interest, and one in which the cause of Homœopathy will be kept well to the front. The local Committee of Arrangements have secured for this meeting the Great Steel Pier, probably the quietest and most ideal spot in the United States for such a meeting. The plans for the entertainment of the Institute are the thought of the entire city, and nothing is being left undone that time or money can do. The greatest seaside resort in the world, a Mecca for health-seekers both winter and summer, a spot where nature and man have vied with each other to do their best, where the very well-to-do or the one with modest income can be supplied with just what they desire, and which I can personally guarantee will be thoroughly satisfactory in rate and in comfort. The profession throughout the East are thoroughly aroused to the interest and importance of this meeting. New England may especially be depended upon to send a good contingent of strong workers, but the same is true of the whole Atlantic coast, and Chicago promises the largest delegation in years, while the West is to be well represented, even from the Pacific Coast.

We recognize the present time as one of crisis in the affairs of men. In medical circles there is unrest, and there never was a time when it was more necessary for our school to present a strong, unbroken front than to-day.

Brother, has Homœopathy done anything for you or yours? Do you, as an honest man, believe in its efficiency as a great law of nature? Yes? Then you owe service and sacrifice that through your love for truth and your fellow-man the truth may be published afar and your fellow-man blessed as you and yours have been. "Set your house in order" and get ready to attend the meeting at Atlantic City.

Fraternally,

BENJ. F. BAILEY, M. D.,
*President.*

---

